# Impact of whole genome sequencing on the care pathway for patients with cancer of unknown primary

**DOI:** 10.1016/j.esmoop.2025.105069

**Published:** 2025-05-08

**Authors:** E. Droogers, Y. Teunissen, A.J. van Puffelen, F.H. Groenendijk, S.E.M. Veldhuijzen van Zanten, A. Wagner, H.M.W. Verheul, D.G.J. Robbrecht

**Affiliations:** 1 Department of Radiology and Nuclear Medicine, Erasmus MC Cancer Institute, University Medical Center Rotterdam, Rotterdam, The Netherlands; 2 Department of Medical Oncology, Erasmus MC Cancer Institute, University Medical Center Rotterdam, Rotterdam, The Netherlands; 3 Department of Pathology, Erasmus MC Cancer Institute, University Medical Center Rotterdam, Rotterdam, The Netherlands; 4 Department of Clinical Genetics, Erasmus MC Cancer Institute, University Medical Center Rotterdam, Rotterdam, The Netherlands

**Keywords:** cancer of unknown primary, whole genome sequencing, whole transcriptome sequencing, actionable alteration

## Abstract

**Background:**

Patients with metastatic disease and no identifiable primary tumor, thus diagnosed with cancer of unknown primary (CUP), typically have a poor prognosis. Tumor DNA sequencing has recently shown promise in identifying the molecular tissue of origin. This study evaluated the value of whole genome sequencing (WGS) in the CUP care pathway, by comparing patient outcomes with a historical control cohort. Also, the value of whole transcriptome sequencing (WTS) was explored.

**Patients and methods:**

A prospective intervention cohort was established of provisional CUP patients (≥18 years of age) who had WGS carried out on metastatic tissue between August 2021 and August 2023. A control cohort was established of CUP patients (≥18 years of age) diagnosed between December 2016 and April 2021 without the availability of WGS. The CUP predicting algorithm was applied to WGS data, and data on definitive diagnosis, molecular actionable alterations [ESMO Scale for Clinical Actionability of molecular Targets (ESCAT) tier 1-3], therapy, diagnostic timelines, and overall survival (OS) were captured.

**Results:**

A total of 159 provisional CUP patients (*n* = 54 intervention cohort, *n* = 105 control cohort) were included. WGS and WTS were successfully carried out in 46 (85%) and 27 patients (50%). A primary tumor diagnosis was established in 76% of the intervention cohort compared with 16% of the control cohort (*P* < 0.001). WGS contributed to a primary tumor diagnosis in 34 patients (63%) and identified an actionable alteration in 34 patients (63%). WTS contributed to a primary tumor diagnosis in three patients (6%). Following WGS, treatment recommendations could be made for 38 patients (70%), of whom 22 started the recommended therapies (58%). The median OS was 11 and 9 months in the intervention and control cohorts, respectively (*P* = 0.345).

**Conclusion:**

Incorporation of WGS into the CUP care pathway is of significant value for diagnosing a primary tumor of origin and contributed to the identification of actionable alterations in the majority of patients.

## Highlights

• Incorporation of WGS into the CUP care pathway led to the identification of a primary tumor in 63% of patients.
• WGS led to the identification of an actionable alteration in 63% of patients.
• Site-specific or alteration-based treatment recommendations could be made for 70% of patients, of whom 58% started therapy.

## Introduction

Cancer of unknown primary (CUP) represents histologically confirmed metastatic disease without evidence of a primary tumor after a comprehensive diagnostic work-up. In recent years, the incidence of CUP has declined, which can be attributed, at least in part, to improved diagnostics.^1^, ^2^, ^3^ CUP, however, still accounts for 1%-2% of all cancer diagnoses worldwide and ranks as the fourth most lethal malignancy.^1^^,^^4^^,^^5^ Unlike patients with cancer from a known primary tumor, two-thirds of patients with CUP are only given best supportive care.^4^^,^^6^^,^^7^ This is presumably the result of poor general condition, typically deteriorated after a long diagnostic trajectory, and lack of therapeutic strategies proven to be beneficial.^2^^,^^7^ The latter could be attributed to CUP being a diverse group of different diseases rather than a single entity. For decades, CUP patients have been treated with empirical chemotherapeutic therapies, mostly platinum or taxane based.^8^, ^9^, ^10^, ^11^ The life expectancy, however, remains poor, with a median overall survival (OS) of several months and a 1-year survival rate of ∼15%.^2^^,^^7^ Therefore, the unmet need for improved diagnostics and appropriate treatment options is high.

According to the contemporary European Society for Medical Oncology (ESMO) guideline for CUP, the existing care pathway for patients with provisional CUP consists of a subset of standard diagnostic examinations supplemented by targeted diagnostics, if necessary (e.g. mammography in female patients).^12^ Experts have speculated whether molecular diagnostics, enabling precision-based medicine in a subset of patients, should be added to this standard routine of care. Molecular diagnostics, such as DNA/RNA sequencing, DNA methylation analysis, and gene expression profiling, as well as algorithmic approaches using molecular data, have been employed aimed at predicting the primary tumor type. These methods have demonstrated high accuracy for tumors of a known primary site and have shown promising results in patients with CUP.^13^, ^14^, ^15^, ^16^, ^17^, ^18^, ^19^, ^20^, ^21^ The CUP predicting algorithm (CUPPA, developed by Hartwig Medical Foundation^20^) has shown to reliably identify the molecular tissue of origin in about two-thirds of CUP patients by integrating whole genome sequencing (WGS) data on tumor type-specific drivers, regional mutational density, and molecular signatures.^20^ CUPPA also has the potential to incorporate data derived from whole transcriptome sequencing (WTS), though this feature has not been validated yet.

Applying WGS for the purpose of primary tumor identification holds the benefit of simultaneously identifying actionable alterations. According to previous literature, approximately one-third of patients with CUP harbor actionable genomic alterations.^10^^,^^22^, ^23^, ^24^, ^25^ Also, since clinical WGS includes germline sequencing of blood DNA, a germline predisposition can be found when applying WGS to tumor tissue, of which patients should be aware before testing. If relevant, consultation with a clinical geneticist should be discussed with the patient and his/her relatives.

Since the spring of 2021, WGS has been reimbursed by the Dutch health care insurance for patients diagnosed with provisional CUP. In the Netherlands, interpretation of outcomes and clinical decision making based on WGS is reserved for dedicated CUP centers. The aim of this single-center study was to evaluate the value of WGS in the CUP care pathway, by comparing lead times, initiated therapies, and patient outcomes with the previous situation without WGS availability. Secondly, we explored the value of WTS, which was not part of the new care pathway, but was available in an experimental setting.

## Patients and methods

### Ethics

Patients with a provisional CUP underwent tumor biopsy from a metastatic lesion for WGS in a standard-of-care setting and consented to the analysis of their clinical and genomic data as part of the available PROGENE trial, which was approved by the medical ethics committee of the Erasmus Medical Center (MC), the Netherlands (MEC 19-0446, 4 August 2021). Consent was waived for the use of clinical data from an archival control cohort of patients without WGS (MEC-2024-0017, 14 March 2024). The study was carried out in accordance with the Dutch Medical Research Involving Human Subjects Act and the Declaration of Helsinki.

### Patient inclusion

All Erasmus MC patients (≥18 years of age) (i) diagnosed with a provisional CUP for whom WGS was applied to a fresh frozen biopsy (or resection material) between August 2021 and August 2023, (ii) with a performance score of ≤2 as defined by the World Health Organization, and (iii) for whom results were discussed in a molecular tumor board (MTB) were prospectively included in the intervention cohort. An archival control cohort consisting of all consecutive Erasmus MC referred patients (≥18 years of age) with a provisional CUP without WGS, between December 2016 and April 2021, was established. Patients with a provisional CUP fulfilling the criteria of one of the favorable subgroups (as per ESMO guidelines) and those with a histologically confirmed neuroendocrine tumor (NET)/carcinoma were excluded in both cohorts.^12^

### The care pathway and clinical parameters

All patients presenting with a provisional CUP received a diagnostic work-up according to the regional applicable care pathway, starting from the time when the cancer was first detected on imaging, including anamnesis, physical examination, laboratory tests (including tumor markers), imaging [at least high-dose computed tomography (CT) scan of the chest and abdomen], diagnostic biopsy (or resection material) of at least one metastatic lesion including an immunohistochemical marker panel guided by the histological pattern, and, if indicated, additional targeted diagnostics (such as mammography, positron emission tomography–CT scan, or consultation by other specialists). In a subset of patients, targeted next-generation sequencing (NGS) was carried out on the metastatic tissue. Following the reimbursement of WGS in 2021, WGS has been integrated into the framework of the care pathway for all CUP patients, requested by a clinical MTB member, after reassessment of the carried out diagnostics.

Data on patients’ medical history, applied diagnostics and timelines, results from WGS and WTS, the definitive diagnosis, treatment recommendation, treatment allocation, referral for genetic counselling, and potential date of death were extracted from the patients’ electronic health records, de-identified, stored in a CASTOR database, and used for baseline characterization and endpoint analyses. The collected data were reviewed by the leading investigator for accuracy. The survival status was captured up to 1 January 2025.

### Whole genome and transcriptome sequencing

For WGS, fresh frozen tumor samples (biopsy or resection material) and blood samples of patients were collected and sent to Hartwig Medical Foundation (Hartwig). Hartwig carried out WGS-based analysis of tumor-normal pairs (90× versus 30× coverage) under International Organization for Standardization (ISO) accreditation, delivering the OncoAct WGS report (see https://oncoact.nl/en/ for details). Experimental and data analysis procedures are extensively validated and described elsewhere.^26^, ^27^, ^28^ The WGS report included relevant data regarding tumor diagnosis, such as tumor purity, molecular tissue of origin (including likelihood of prediction), mutation status, actionability of identified molecular alterations, and potentially matching standard of care and experimental treatment options. During a weekly organized MTB, consisting of medical oncologists, clinical geneticists, pathologists, clinical scientists in molecular pathology, and a pulmonologist, the WGS report was discussed to establish a definitive integrated diagnosis and, when applicable, to determine allocation to treatment.

In addition to WGS, WTS was applied in the context of the PROGENE trial, which enabled exploration of the added value of WTS in CUP patients. WTS results were also discussed in the MTB meetings.

### CUPPA

CUPPA is a tool that uses a range of genomic features to match a sample’s profile with samples from known primary tumors and has been applied to WGS.^20^ This prediction tool was developed and trained by Hartwig Medical Foundation by combining tumor type-specific drivers, regional mutational density, and mutational profile characteristics on a large WGS cohort of >4000 histopathologically confirmed primary tumor samples. After internal and external validation (on 254 patients with tumor WGS from an independent cohort), the algorithm was deemed feasible and valid, with predictive precision reaching 95% with a similarity likelihood threshold of 0.8. Further details are available in Schipper et al.^20^

### Clinical value of WGS

The clinical value of WGS was determined based on the number of CUP patients in whom WGS contributed to a primary tumor diagnosis and/or identification of actionable alterations. In our study, a high-confidence prediction (>0.8) was considered decisive for the primary tissue of origin. In case of a low-confidence prediction (<0.8), the WGS profile was defined as a potential supportive outcome. There was no threshold at which WGS was considered definitively inconclusive. Actionable alterations included DNA alterations according to the ESMO Scale for Clinical Actionability of molecular Targets (ESCAT) tier 1 (definition: alteration–drug match is associated with improved outcome in clinical trials), tier 2 (definition: alteration–drug match is associated with antitumor activity, but magnitude of benefit is unknown), and tier 3 [definition: alteration–drug match suspected to improve outcome based on clinical trial data in other tumor type(s) or with similar molecular alteration].^29^ Actionable alterations were divided into genomic alterations [such as *KRAS* and *PTEN* mutations] and genomic markers [such as tumor mutational load/burden (TML/TMB), homologous recombination deficiency (HRD), and microsatellite instability (MSI)].

### Clinical value of WTS

The clinical value of WTS was determined based on the number of CUP patients in whom the WTS results confirmed or contributed to the primary tumor diagnosis and had impact on treatment recommendation.

### (Statistical) data analysis

The χ^2^ test was carried out to compare the incidence of a primary tumor diagnosis and treatment initiation between both patient cohorts and reported by odds ratios (ORs) along with 95% confidence intervals (CIs). A Mann–Whitney *U* test was carried out to evaluate whether lead times during diagnostic work-up differed per patient cohort. The median OS was calculated and differences in 1-year OS were evaluated using the Kaplan–Meier analysis with log-rank tests. A Cox proportional hazards regression model was used to adjust for potential confounders, including age, gender, year of CUP diagnosis, systemic therapy, and performance status. Results were reported as hazard ratios (HRs) with 95% CIs. Lead times and OS were all measured from the date of first imaging showing metastatic disease. Normality of the data was determined using the Kolmogorov–Smirnov *Z* test for continuous variables and reported as mean and standard deviation for normally distributed variables and as median and interquartile range (IQR) for non-normally distributed variables. Categorical variables were reported as frequencies and percentages. Statistical analysis was carried out using the Statistical Package for the Social Sciences (SPSS) version 28.0.1.0.1 (IBM Corporation, Armonk, NY). All statistical values were two-sided and considered significant at *P* < 0.05.

## Results

### Patient characteristics

A total of 159 patients were included in this study, comparing 54 patients in the intervention cohort with 105 patients in the control cohort. Patient characteristics and histopathology results are summarized in Supplementary Tables S1 and S2, available at https://doi.org/10.1016/j.esmoop.2025.105069. The median age at diagnosis was 66 (IQR 55-70) and 64 (IQR 57-71) years in the intervention and control cohorts, respectively. In both cohorts, most metastases were found in the lymph nodes (61% and 67%) followed by the liver (41% and 33%).

In the intervention cohort, WGS was successfully carried out in 46 of 54 patients (85%). The reasons for unsuccessful sequencing were low tumor purity (7/8) or insufficient material (1/8). WTS was carried out on an exploratory basis in 27 of 54 (50%) patients. In the remaining patients, WTS was not carried out due to the temporary unavailability of WTS at the sequencing center, insufficient quality of tissue, or other technical issues.

### Definitive diagnosis

**Figure 1:**
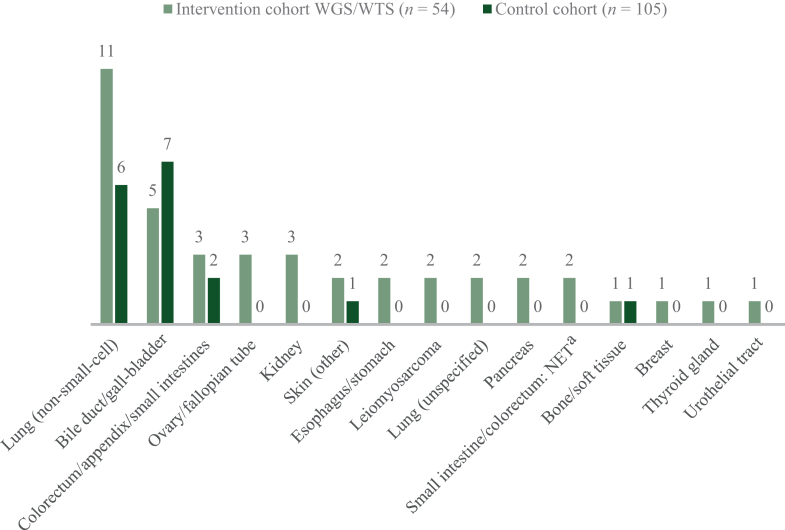
**Primary tumor diagnosis.** Values are presented as absolute numbers. NET, neuroendocrine tumor; WGS/WTS, whole genome/transcriptome sequencing. ^a^Since NET diagnoses were confirmed after WGS, these patients were not excluded.

At the end of the diagnostic work-up, significantly more patients in the intervention cohort were diagnosed with a (molecular) primary tumor compared with the control cohort (76% and 16%; OR 16.3, 95% CI 7.3-36.8, *P* < 0.001). The most common primary tumor diagnoses were non-small-cell lung carcinoma (20% and 6%) and cancer originating from the bile duct or gall-bladder (9% and 7%, Figure 1). For 34 out of 54 (63%) patients in the intervention cohort, the WGS outcome was either conclusive (confidence prediction ≥0.8) in 19 patients or supportive (confidence prediction <0.8) in 15 patients (Supplementary Figure S1, available at https://doi.org/10.1016/j.esmoop.2025.105069). In 20 patients, the WGS results were inconclusive for tissue of origin, of whom 6 patients eventually received a primary tumor diagnosis after additional diagnostics over time.

In nine patients, WTS confirmed the primary tumor diagnosis already identified through WGS. In three patients, WTS was supportive for the primary tumor diagnosis. In 15 patients, the WTS outcomes were not of value (Supplementary Table S2, available at https://doi.org/10.1016/j.esmoop.2025.105069).

In the intervention cohort, diagnoses of leiomyosarcoma (*n* = 2), NET (*n* = 2), and thyroid cancer (*n* = 1) were established following WGS/WTS results, with pathology assessment already suggesting, yet not confirming, these diagnoses in four of five patients.

### Actionable DNA alterations

Among 27 patients (50%) in the intervention cohort, WGS revealed 31 actionable genomic alterations. Additionally, genomic markers (e.g. TML, MSI, HRD) were identified in 22 patients (41%), resulting in a total of 34 patients (63%) with an actionable target (of whom 6 had ESCAT tier 1 alterations, Supplementary Table S3, available at https://doi.org/10.1016/j.esmoop.2025.105069). *KRAS* alterations were the most frequent genomic alterations present (*n* = 11), followed by *PTEN* alterations (*n* = 5) and *BRAF* V600E mutations (*n* = 4). No driver fusions were detected. High TML was found in 19 of 54 (35%) patients. Actionable alterations are summarized in Supplementary Table S3, available at https://doi.org/10.1016/j.esmoop.2025.105069.

### Treatment

After concluding the definitive diagnosis, systemic treatment was initiated in 59% of patients in the intervention cohort and 37% of patients in the control cohort (OR 2.5, 95% CI 1.3-4.8, *P* = 0.008). An overview of the initiated treatments is shown in Table 1. Treatment recommendations based on WGS could be made for 38 patients (70%). Of these, the recommended treatment was initiated in 22 patients (58%): site-specific treatment (*n* = 18) or alteration-based treatment [*n* = 1 regular treatment (ESCAT 1) and *n* = 3 clinical trial]. Reasons for not starting treatment included death, deteriorated clinical condition, and patient’s wishes (Table 1).

**Table 1:** Overview of treatment initiation

	Intervention cohort (WGS/WTS) (*n* = 54)	Control cohort (*n* = 105)	OR (95% CI), *P* value
Treatment started after definitive diagnosis			1.9 (1.0-3.7), *P* = 0.066
Yes	36 (67%)	53 (51%)	
No	18 (33%)	52 (49%)	
Type of treatment			
Systemic	23 (43%)	33 (31%)	
Surgery	0 (0%)	2 (2%)	
Radiotherapy	4 (7%)	12 (11%)	
Systemic/surgery	3 (6%)	1 (1%)	
Systemic/radiotherapy	6 (11%)	5 (5%)	
Systemic cancer therapy	32 (59%)	39 (37%)	2.5 (1.3-4.8), *P* = 0.008
Chemotherapy	13 (24%)	31 (30%)	
Immunotherapy	7 (13%)	4 (4%)	
Tyrosine kinase inhibitors	2 (4%)	0 (0%)	
Combination therapy	9 (17%)	4 (4%)	
Other^a^	1 (2%)	0 (0%)	
Treatment started within the context of clinical trial	3^b^ (6%)	14^c^ (13%)	
Rationale for the absence of treatment			
Death	3 (6%)	3 (3%)	
Deteriorated clinical condition	8 (15%)	32 (30%)	
Patient’s wishes	5 (9%)	11 (10%)	
Missing data	2 (4%)	6 (6%)	

Values are presented as *n* (%).

CI, confidence interval; OR, odds ratio; WGS/WTS, whole genome/transcriptome sequencing.

a Peptide receptor radionuclide therapy.

b NCT03114319 (*n* = 1), DRUP study (https://drupstudy.nl/) (*n* = 2).

c NCT02442531 (*n* = 3), NCT06132191 (*n* = 1), NCT02304393 (*n* = 1), NCT03498521 (*n* = 5), NCT03668119 (*n* = 1), NL-OMON29351 (*n* = 2), DRUP study (https://drupstudy.nl/) (*n* = 1).

### Lead times diagnostic work-up

**Figure 2:**
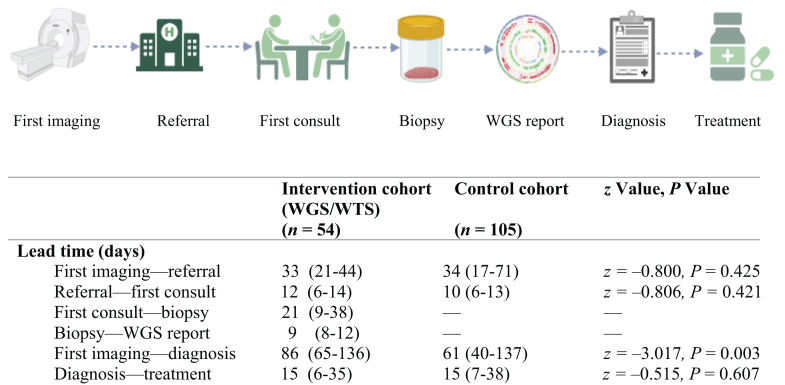
**Timelines for diagnostic work-up.** Values are presented as median (IQR). IQR, interquartile range; WGS/WTS, whole genome/transcriptome sequencing.

No significant difference between lead time from first imaging showing metastatic disease to referral to the Department of Medical Oncology at Erasmus MC was observed between the two cohorts (33 days, IQR 21-44 days and 34 days, IQR 17-71 days, *z* = −0.800, *P* = 0.425). A new tumor biopsy was carried out at a median of 21 days (IQR 9-38 days) after first consultation, followed by WGS with a median turnaround time of 9 days (IQR 8-12 days). The lead time from first imaging (either externally or at Erasmus MC) to definitive diagnosis (either a primary tumor diagnosis or a confirmed CUP diagnosis) was 86 days in the intervention cohort (IQR 65-136 days) and 61 days in the control cohort (IQR 40-137 days, *z* = −3.017, *P* = 0.003). Treatment, if applicable, was started at a median of 15 days after definitive diagnosis in both cohorts (Figure 2).

### Survival data

**Figure 3:**
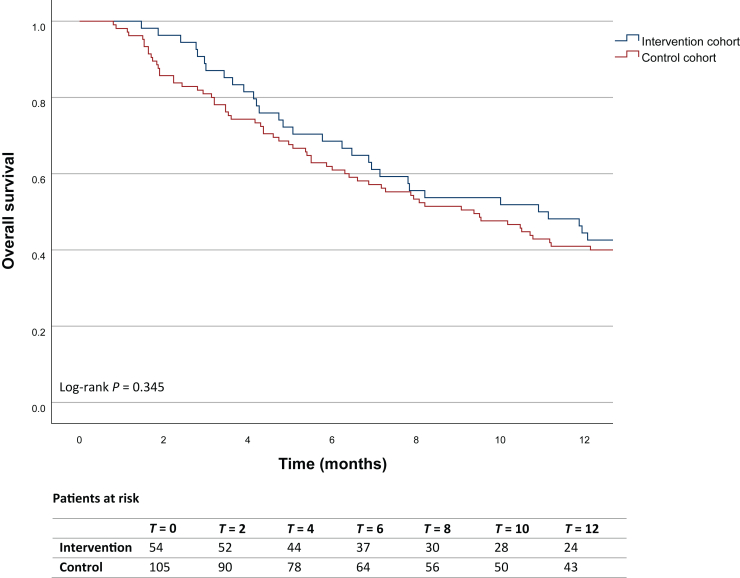
**Kaplan–Meier analysis showing the 1-year survival of patients in the intervention (WGS/WTS) and control cohorts.** There are no censored cases during the 12-month time frame. WGS/WTS, whole genome/transcriptome sequencing.

**Figure 4:**
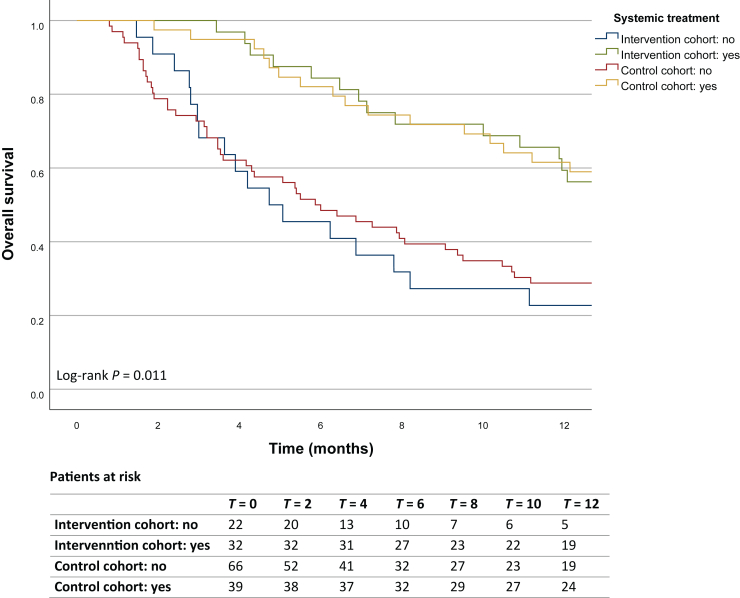
**Kaplan–Meier analysis showing the 1-year survival of patients with and without systemic cancer treatment in the intervention (WGS/WTS) and control cohorts.** There are no censored cases during the 12-month time frame. WGS/WTS, whole genome/transcriptome sequencing.

The Kaplan–Meier survival analysis showed a 1-year survival rate of 44% (95% CI 31.1% to 57.8%) in the intervention cohort, compared with 41% (95% CI 38.0% to 44.0%) in the control cohort. The median OS was 11 months (95% CI 6.0-15.8 months) and 9 months (95% CI 6.4-12.3 months), respectively. The difference in survival between the two cohorts was not statistically significant (log-rank test: χ^2^ = 0.892, *P* = 0.345, Figure 3). After adjusting for covariates, the difference in survival remained non-significant (HR 0.844, 95% CI 0.51-1.40, *P* = 0.512, Supplementary Table S4, available at https://doi.org/10.1016/j.esmoop.2025.105069). The 1-year survival rate of patients treated with systemic cancer therapy was 59% (95% CI 32.5% to 86.2%) for the intervention cohort and 62% (95% CI 33.1% to 90.0%) for the control cohort, while the 1-year survival rate with only best supportive care was 23% (95% CI 2.1% to 43.3%) and 29% (95% CI 7.8% to 49.8%), respectively (Figure 4).

### Referral for genetic counselling

In the intervention cohort, nine patients (17%) were referred for counselling by a clinical geneticist, compared with three patients (3%) in the control cohort. In the intervention cohort, referral was (at least partially) based on WGS findings in eight of nine patients. Additionally, family medical history and patient’s age at cancer diagnosis were reasons for referral to the clinical geneticist in the other patient (1/9). Of these nine patients, six were counselled by a clinical geneticist from Erasmus MC, of whom one had a newly discovered germline mutation. In the control cohort, referrals were based on family medical history and/or patient’s age at cancer diagnosis (3/3), and none of them appeared to have germline mutations.

## Discussion

This study evaluated the value of WGS incorporation in the CUP care pathway. Results show a significant increase in the establishment of a primary tumor diagnosis compared with the control cohort (76% versus 16%). WGS contributed to the primary tumor diagnosis in 63% of patients, with a high-confidence prediction of the molecular tissue of origin in >50% of these patients. In line with our results, previous studies have also demonstrated the positive predictive capacity of molecular-genetic diagnostics in determining the tissue of origin, with rates varying from 41% to 89%.^14^^,^^15^^,^^19^, ^20^, ^21^^,^^30^^,^^31^ Notably, two prior studies that also applied CUPPA assigned a primary tumor diagnosis to 46 of 72 (64%) and 56 of 73 (77%) CUP cases, respectively.^20^^,^^32^

In addition to deep learning classifiers for WGS data, classifiers have also been developed for panel data, such as NGS-based approaches. For instance, Moon et al. developed the NGS-based primary cancer-type classifier (OncoNPC) and reported high-confidence predictions (>0.9) in 41% of CUP patients.^19^ While limited data are available on the direct comparison of panel sequencing and WGS, Rebello et al. reported that panel sequencing alone provided diagnostic insight in 33% of 58 cases, whereas this increased to 54% when using WGS/WTS. They also showed that most single nucleotide variants and structural variants were only detected by WGS/WTS, and that specific mutation signatures, such as SBS4 for tobacco smoking, were missed by panel sequencing due to the lower number of variants detected.^32^ These findings highlight that while panel sequencing can aid in primary tumor detection, WGS-based prediction tools offer greater accuracy by incorporating the entire genome. This is further supported by Jiao et al. who underscored how patterns of somatic passenger mutations can accurately predict 24 common cancer types, while adding driver mutation data reduced the accuracy of their deep learning model for primary tumor prediction.^13^

Noteworthily, while primary tumor diagnoses based on genome sequencing rely on a molecular tissue of origin with a certain degree of certainty, this does not exclude the possibility that cancers diagnosed ‘molecularly’ may have different biological characteristics compared with those identified through regular diagnostics.

Besides the establishment of a definitive diagnosis, actionable alterations were identified in 34 patients (63%), which is slightly more than the 47% reported by Schipper et al.^20^ Identification of actionable alterations allows for selection of patients who might benefit from targeted therapies, both those with confirmed CUP and those whose diagnosis changes to a known primary origin. Despite the high number of patients with a primary tumor diagnosis and/or identification of an actionable alteration, only 22 patients received WGS-guided treatment. Treatment options based on WGS were available for 16 other patients, but were not initiated due to death, deteriorated clinical condition, or expressed patient’s wishes. In the control cohort, more patients participated in a clinical trial, which can be attributed to a stronger reliance on last-resort early clinical trials.

Given the retrospective nature of this study, the small sample size, and the lack of detailed information regarding therapy, evaluating survival outcomes with and without WGS and the subsequent therapy administered was not the primary intent of this study. Nevertheless, we show a limited absolute survival difference in a median OS of 2 months (11 versus 9 months; non-significant) between the intervention and control cohorts. A previous meta-analysis, consisting of 453 patients who received site-specific therapy and 660 patients who received empiric chemotherapy, revealed that site-specific therapy was not significantly associated with improved progression-free survival (PFS, HR 0.93, 95% CI 0.74-1.17, *P* = 0.534) and OS (HR 0.75, 95% CI 0.55-1.03, *P* = 0.069), compared with empiric therapy.^33^ Moreover, the GEFCAPI04 trial randomized patients with CUP to standard cisplatin/gemcitabine treatment or site-specific therapy (‘gene expression test followed by à la carte treatment according to the suspected primary’) but did not find improved PFS with the targeted approach. However, results from this study were hampered by a significant proportion of patients receiving platinum-based chemotherapy as site-specific therapy in the intervention arm, and the results cannot exclude benefit from contemporary therapies not available at the time of the study conduct.^34^

The recently published CUPISCO trial showed a very modest PFS benefit of 1.7 months in patients treated with molecularly guided therapy following three cycles of platinum-based chemotherapy versus patients in whom platinum-based chemotherapy was continued.^35^ CUPISCO does not address the benefits of site-specific therapy since this study did not aim to determine the presumptive primary tumor. The true value of site-specific therapy can only be determined through a randomized trial after defining the (molecular) primary tumor of origin. Therefore, prospective clinical trials are warranted to provide evidence on the impact on survival outcomes of WGS and subsequent site-specific treatments. Also, the impact on cost–benefit should be assessed in future trials.

Beyond the potential to improve patient survival, identifying the molecular tissue of origin and thereby reclassifying the diagnosis from CUP to a cancer from a known primary origin could significantly enhance patients’ mental well-being. Hyphantis et al. showed that CUP patients are known to experience more psychological distress than patients with metastatic disease of a known primary.^36^ Identification of the primary tumor could offer mental relief to these patients and their relatives. However, WGS increases the likelihood of coincidentally finding a germline predisposition to cancer (17% in our intervention cohort), which may induce stress among patients or relatives. Nevertheless, such findings may also benefit relatives at risk, as they can take preventive measures. Pre-test information about the risk of incidental findings, and counselling by a clinical geneticist when applicable, is therefore essential.^37^

Furthermore, the diagnostic process for CUP patients remains time consuming, with our intervention cohort showing a delay of 25 days in the establishment of a definitive diagnosis due to the need for fresh tumor biopsies and WGS analysis.^26^ Prolonged diagnostic work-up often leads to clinical deterioration, causing a low performance status at the time of therapy initiation.^38^ A shorter diagnostic timeline is therefore assumed to increase the proportion of patients fit for therapy. This highlights the importance of early integration of WGS into the CUP care pathway and emphasizes the need for enhanced intercollegiate collaboration between regional hospitals and CUP centers.

Lastly, the clinical value of WTS was evaluated. In most patients, WGS alone was sufficient to identify the molecular tissue of origin, suggesting that, at present, WTS does not offer clear and cost-effective additional value in the context of CUP.

This study offers a real-time evaluation of WGS incorporation into routine clinical care for CUP patients; however, some limitations must be acknowledged. Firstly, this study was not powered to detect an OS benefit from WGS incorporation. Secondly, the retrospective nature of the control cohort introduces uncertainty, particularly regarding treatment initiation and follow-up. Furthermore, the data do not account for CUP patients who were not referred to a CUP center and for whom WGS is not carried out. In addition, 15% of WGS analyses were unsuccessful due to low tumor purity or insufficient material, for which sequencing analysis on liquid biopsies may be a promising alternative.^32^^,^^39^, ^40^, ^41^

In conclusion, incorporating WGS into the CUP care pathway provides substantial clinical value, resulting in a 63% reclassification from CUP to a cancer with a known primary site and the identification of actionable alterations in 63% of patients, enabling most to receive site-specific or molecularly targeted therapy. Prospective clinical trials are warranted to provide high-level evidence on the impact on survival outcomes of WGS-informed change in diagnosis and treatment decisions.

## Glossary

**Provisional CUP:** Metastatic epithelial or neuroendocrine malignancy identified on the basis of histology or cytology, with no primary site detected despite a selected initial screen of investigations, before specialist review and possible further specialized investigations.^42^

**Confirmed CUP:** Metastatic epithelial or neuroendocrine malignancy identified on the basis of final histology, with no primary site detected despite a selected initial screen of investigations, specialist review, and further specialized investigations as appropriate.^42^
