# Supplementary material for: Impact of whole genome sequencing on the care pathway for patients with cancer of unknown primary
Source: ESMO Open. 2025 May 8;10(5):105069. doi: 10.1016/j.esmoop.2025.105069 (PMC12136782; doi:10.1016/j.esmoop.2025.105069)
Supplement: Supplementary Figure S1 [file mmc1.docx]

**Supplementary Figure S1** Contribution of WGS/WTS to definitive diagnosis

In 3 patients, WTS was not concordant with the WGS results, but considered supportive for the primary tumor diagnosis. Patient 1: leiomyosarcoma, with WTS predicting leiomyosarcoma as the primary tumor (41%). WGS – available 1 week prior to the WTS results - suggested a bone/soft tissue tumor, but was at the time considered too aspecific. Patient 2: bile duct/gallbladder cancer, with WTS predicting bile duct/gallbladder as the primary tumor (67%). WGS identified BAP1 and BPRM1 alterations, which were considered supportive for the diagnosis. Patient 3: NSCLC, with WTS predicting NSCLC as the primary tumor (32%). WGS suggested SCLC and was considered supportive. In 9 patients, WTS confirmed the primary tumor diagnosis already identified through WGS

CUP = cancer of unknown primary. WGS/WTS: whole genome/transciptome sequencing.
