# Supplementary material for: Impact of whole genome sequencing on the care pathway for patients with cancer of unknown primary
Source: ESMO Open. 2025 May 8;10(5):105069. doi: 10.1016/j.esmoop.2025.105069 (PMC12136782; doi:10.1016/j.esmoop.2025.105069)
Supplement: Supplementary Table S1 [file mmc2.docx]

**Supplementary Table S1** Patient characteristics

| Characteristics | Intervention cohort (WGS/WTS)  *(n=54)* | Control cohort  *(n=105)* |
| --- | --- | --- |
| Sex  *Male*  *Female* | 25 (46%)  29 (54%) | 51 (49%)  54 (51%) |
| Age | 66 (55 -70) | 64 (57 – 71) |
| Site of metastasis  *Lymph nodes*  *Liver*  *Peritoneum*  *Bone*  *Lung*  *Other*  *Central nervous system* | 33 (61%)  22 (41%)  14 (26%)  14 (26%)  17 (31%)  15 (28%)  0 (0%) | 70 (67%)  35 (33%)  20 (19%)  27 (26%)  32 (30%)  38 (36%)  3 (3%) |
| Performance score  *WHO 0*  *WHO 1*  *WHO 2*  *WHO 3 or higher*  *Unknown* | 10 (19%)  35 (65%)  7 (13%)  0 (0%)  2 (4%) | 13 (12%)  56 (53%)  19 (18%)  9 (9%)  8 (8%) |
| Endoscopic evaluation  *Gastroscopy*  *Colonoscopy* | 28 (52%)  21 (39%) | 54 (51%)  36 (34%) |
| Prior treatment  Overall^a^  *Surgery*  *Radiotherapy*  *Systemic*  *Surgery/radiotherapy*  *Surgery/systemic*  *Radiotherapy/systemic*  *Surgery/radiotherapy/systemic*  *Hyperthermia* | 21 (39%)  0 (0%)  5 (9%)  9 (17%)  3 (6%)  1 (2%)  1 (2%)  2 (4%)  0 (0%) | 37 (35%)  4 (4%)  13 (12%)  11 (10%)  4 (4%)  1 (1%)  2 (2%)  1 (1%)  1 (1%) |

Values are presented as median (IQR) and n (%). WGS/WTS: whole genome/transciptome sequencing.

^a^A total of 28 patients received systemic cancer therapy: chemotherapy n=24,

chemotherapy + immunotherapy n=3, somatostatin analogue n=1
