# Supplementary material for: Impact of whole genome sequencing on the care pathway for patients with cancer of unknown primary
Source: ESMO Open. 2025 May 8;10(5):105069. doi: 10.1016/j.esmoop.2025.105069 (PMC12136782; doi:10.1016/j.esmoop.2025.105069)
Supplement: Supplementary Table S3 [file mmc4.docx]

**Supplementary Table S3** Actionable alterations identified through WGS

|  | Actionable alteration | Specification |
| --- | --- | --- |
| *Genomic alteration* | *ERBB2 (n=3)* | Amplification *(n=3)* |
|  | *MET (n=1)* | *MET p.H1112Y (n=1)* |
|  | *KRAS (n=11)* | *KRAS p.G12R (n=2)*  *KRAS p.G12C (n=2)*  *KRAS p.G12V (n=1)*  *KRAS p.G12D (n=1)*  *KRAS p.Q61L (n=2)*  *KRAS p.Q61H (n=1)*  *KRAS p.G13D (n=2)* |
|  | *BRAF (n=4)* | *BRAF p.V600E (n=4)* |
|  | *MAP2K1 (n=1)* | *MAP2K1 p.C121S (n=1)* |
|  | *NF1 (n=2)* | Homozygous disruption *(n=1)*  *NF1 p.? (n=1)* |
|  | *PTEN (n=5)* | *PTEN p.A39V (n=1)*  *PTEN p.N12_R14del (n=1)*  Homozygous disruption *(n=1)*  Partial bi-allelic loss *(n=2)* |
|  | *PIK3CA (n=2)* | *PIK3CA p.N345K (n=1)*  *PIK3CA p.E453K (n=1)* |
|  | *BRCA 1/2 (n=2)* | *BRCA1 p.E1011 + BRCA1 p.? (n=1)*  *BRCA2 p.Q2354E + BRCA2 p.T2834I (n=1)* |
| *Genomic marker* | High TMB and/or TML (n=19) | $ |
|  | Microsatellite instability (*n=2*) | $ |
|  | Homologous recombination deficiency (*n=5*) | $ |

­­­

$ = not applicable

WGS = whole genome sequencing
