# Supplementary material for: Impact of whole genome sequencing on the care pathway for patients with cancer of unknown primary
Source: ESMO Open. 2025 May 8;10(5):105069. doi: 10.1016/j.esmoop.2025.105069 (PMC12136782; doi:10.1016/j.esmoop.2025.105069)
Supplement: Supplementary Table S4 [file mmc5.docx]

**Supplementary Table S4** Cox proportional hazards regression analysis

|  | HR (hazard ratio) | 95% CI | *p*-value |
| --- | --- | --- | --- |
| Cohort (WGS/WTS) | 0.844 | 0.509 - 1.400 | 0.512 |
| Age (per year) | 1.017 | 1.002- 1.033 | 0.024 |
| Gender (female) | 0.696 | 0.490 - 0.988 | 0.042 |
| Year of CUP diagnosis | 1.098 | 0.977 - 1.233 | 0.117 |
| Systemic therapy (yes) | 0.724 | 0.497 - 1.053 | 0.091 |
| WHO 1 | 2.103 | 1.248 – 3.545 | 0.005 |
| WHO 2 | 3.485 | 1.847 – 6.575 | <0.001 |
| WHO >2 | 18.724 | 7.106 – 49.338 | <0.001 |
| WHO unknown | 4.027 | 1.585 – 10.227 | 0.003 |

CI = Confidence Interval, HR = Hazard Ratio; WHO – World Health Organization performance score. WGS/WTS = whole genome/transciptome sequencing

Omnibus test: *p*<0.001
